# Mouth breathing in children with learning disorders

**DOI:** 10.5935/1808-8694.20130111

**Published:** 2015-10-08

**Authors:** Giovana Serrão Fensterseifer, Oswaldo Carpes, Luc Louis Maurice Weckx, Viviane Feller Martha

**Affiliations:** a Medical Student - Medical School - Pontifical Catholic University of Rio Grande do Sul (PUCRS) ATM 2014.; b PhD in Medicine - PUCRS (Head of the Facial Aesthetic Surgery Division - Department of Otolaryngology and Head and Neck Surgery - PUCRS).; 3† PhD in Otorhinolaryngology - Federal University of São Paulo (in memoriam).; d PhD in Medicine - Federal University of Rio Grande do Sul (UFRGS). Preceptor of Otorhinolaryngology - PUCRS. Otorhinolaryngology Service - São Lucas Hospital - Pontifical Catholic University of Rio Grande do Sul (PUCRS).

**Keywords:** adenoids, learning disorders, mouth breathing, nasal obstruction

## Abstract

Given the importance of studying the causes of learning disorders, we designed this case-control study to assess the nasal cavity volume, pharyngeal and palatine tonsils in children with and without learning disabilities.

**Method:**

A total of forty-eight children were enrolled in the study: twenty-four coming from the Center for Evaluation and Early Stimulation (CADEP), in which the criterion is the school failure of at least two consecutive years; and twenty-four students with normal learning - which made up the control group. The children were submitted to ENT examination (history, physical examination) and specific tests (acoustic rhinometry, *cavum* radiography).

**Results:**

The results showed that students with learning disabilities have a higher prevalence of pharyngeal tonsil hypertrophy: *p* < 0.001, and palatine tonsil hypertrophy: *p* < 0.001. The average volume of the nasal cavities showed no statistically significant association with learning difficulties (*p* = 0.75).

**Conclusion:**

Based on this study, we concluded that children with adenotonsillar hypertrophy have more learning difficulties when compared to children without such hypertrophy.

## INTRODUCTION

According to the National Joint Committee on Learning Disabilities, the term learning disability is a general term that refers to a heterogeneous group of disorders manifested by difficulties in acquiring and using attention, speaking, reading and writing, reasoning and mathematical skills^1^.

The term learning refers to the complex frameworks of language, cognitive, physiological, psychological and sociological phenomena associated with being human. All these factors are involved in the learning mechanism; however, the pathways through which they interact have not been entirely understood^2^.

School failure is a phenomenon that affects the entire Brazilian society, regardless of socioeconomic class - a complex problem that stems from multiple causes. For every 100 children who enter the first grade, only 12 reach the eighth grade. This scenario is worsened in the lower income strata, in which about 80% of the children fail school^3^.

Despite how nasal obstruction alone in its various degrees of involvement impacts learning is not enough documented, it is known that children with nasal obstruction due to hypertrophy of the pharyngeal tonsils may show some degree of hypoxemia^4^. Although the relationship of nasal obstruction with learning is not fully understood, we know that chronic nasal obstruction may precipitate recurrent infections of the upper and lower respiratory tract, and this is an important factor in the development of cardiopulmonary syndrome. Therefore, the consequences of respiratory disorders in learning, in its various degrees, are not fully appreciated^5^.

In the literature, the published results regarding the association of oral breathing and learning disability are scarce and controversial. In an attempt to better understand this relationship, we designed a prospective study aiming to assess the size of the pharyngeal and palatine tonsils and the nasal cavity volume, and to correlate these data with poor school performance.

## METHOD

This study was approved on its ethical and methodological issues by the Scientific Committee of the Medical School and São Lucas Hospital in 1999. All patients who agreed to participate in this study signed the Informed Consent Form, through their legal representatives, for a anonymously use of their information in scientific publications. Our study has a case-control design. The inclusion criteria were children aged 8 to 12 years with learning disabilities and normal hearing, who were selected by the Disability Evaluation Center and Early Stimulation (CADEP). This agency belongs to the Department of Education and Culture of the State of Rio Grande do Sul and is responsible for identifying and handling potential problems involving these children, which could influence their school performance.

The students were referred to this institution from the public schools of greater Porto Alegre, RS, Brazil. The criterion used by teachers for referral to CADEP is school failure, which should be of at least two consecutive years. The initial CADEP approach starts with neuropediatric, psychological and social assessments.

As a control, we used children of the same age who had normal school performance. These students came from the Paulina Moresco public school, located in Porto Alegre, which has a high prevalence of children referred to CADEP.

The sample had two groups:

• Group I - Children with learning difficulties - 24 children between 8 and 12 years of age, of both genders, referred to CADEP because of a learning disability, with normal neuropediatric, psychological, social and clinical assessments;
• Group II - Children without learning disabilities - 24 children between 8 and 12 years of age, of both genders, from the Paulina Moresco School, with normal school performance.

The exclusion criteria were: neuropediatric disorder - diagnosed through physical examination (motor test), and/or complementary tests (EEG and CT scan), and altered psychological evaluation, conducted by a CADEP psychologist. The evaluation consists of interviews with the child's guardians and with the child to assess whether their intellectual development matches their chronological age. For this evaluation we used a standardized test called “Game Time” (Johnson, 1997). This test, which was carried out with the participation of the mother, is a play session with methodological tools as play dough and drawings. This activity aims to study, through verbal and nonverbal language, small signs of possible causes of change in the child's behavior.

We also excluded those children who had social disorders, which is characterized by the presence of hunger and extreme poverty. We excluded children with air conduction and sensorineural hearing loss.

Both groups were submitted to nasal breathing assessment according to the “Protocol for assessment of children with learning disabilities”. Below is a description of how this protocol defines nasal obstruction, apnea and nasal secretion. The protocol also defines criteria for mouth exam, anterior rhinoscopy, *cavum* X-ray and echo-rhinometry, as per explained below.

The ENT history was taken from the child's guardian to assist with the answers.

We defined as having nasal obstruction those patients who breathed through their mouths more than 50% of the time for at least three years.

Snoring was classified as present when there was a history of snoring more than four nights a week, for at least three years.

Apnea was classified as present when a family member of the patient reported the patient stopped breathing for about 15 seconds, also for at least three years. We did not order a polysomnography and the apnea was only clinically diagnosed.

Nasal discharge was characterized as present if it occurred for more than ten consecutive days, and had begun at least three years before.

The ENT examination, done at the office, assessed tonsil size - which were classified as normal (grade I) - it did not extend beyond the line of the anterior pillar; moderate hypertrophy (grade II) - where it extended to beyond the anterior pillar; severe hypertrophy (grade III) - when they occupied more than 50% of the oropharynx, but did not touch the midline and, hypertrophy causing total obstruction (grade IV) - when the tonsils touched each other in the midline of the oropharynx^6^.

On anterior rhinoscopy we assessed the size of the turbinates, classifying them as hypertrophied when the inferior turbinates were impacted against the nasal septum, and normal, when they were outside these parameters.

All children were subjected to radiological examination of the nasopharynx to assess pharyngeal tonsil size. The nasopharynx (*cavum*) was defined as the area between a line parallel to the upper surface of the palatine bone all the way to the anterior wall of the occipital bone (adapted from Fugioka et al.) in a side view at 15 cm from the film. The nasopharyngeal space comprising this line was divided into four parts and analyzed according to the degree of adenoid growth:

• Stage I - Atrophic pharyngeal tonsil;
• Stage II - Normal pharyngeal tonsil;
• Stage III - Hypertrophic pharyngeal tonsil; and
• Stage IV - Total obstruction^7^.

To establish the anatomical configuration of the nasal cavity and nasopharynx, all the students underwent echo-rhinometry. This test was performed with the patient seated, and the device was calibrated for this purpose. The parameter evaluated was the nasal cavities' volume, in cubic centimeters.

The statistical analysis of the qualitative variables (pharyngeal and palatine tonsils) was performed using nonparametric tests. The quantitative variable (echo-rhinometry) study was carried out using parametric tests in view of its normal frequency and homogeneous variance. All comparisons were performed for independent samples (Mann-Whitney test and *t*-test). For statistical purposes, we defined *p* < 0.5 as the significance criterion.

## RESULTS

Between 1998 and 2000, 45 school children from schools in poor socioeconomic conditions from the city of Porto Alegre, RS, underwent hearing and nasal breathing assessment. The average age of both groups was 9.1 years, 42% females in group I (children with learning difficulties) and 33% females in Group II (children without such difficulties). The mean number of repeated years in school was 2.8 years. The results concerning nasal function and presence or absence of obstruction can be found on Table 1.

**Table 1:** Comparison of characteristics between the groups with learning difficulties (w/LD) and without learning difficulties (wo/LD).

		Groups		
Characteristic	Total n = 48	w/LD n =24	wo/LD n = 24	P
Age (years)	9.1 ±2.0	9.0 ± 2.0	9.2 ± 2.0	>0.99
Females (%)	18 (37.5)	10(42.0)	8 (33.3)	0.77

Nasal breathing (%)				

Nasal obstruction	41.4	54.2	28.6	0.14
With snoring	44.4	45.8	42.9	>0.99
With apnea	19.7	25.0	14.3	0.46
Nasal secretion	56.9	70.8	42.9	0.08

Volume (cm^3^)				

Left nostril	3.8 ± 1.0	3.9 ± 1.0	3.7 ± 0.9	0.47
Right nostril	4.1 ± 1.1	3.8 ± 0.8	4.3 ± 1.4	0.14
Both (mean)	4.0 ± 1.1	3.9 ± 0.9	4.0 ± 1.2	0.75

Hypertrophy degree of the palatine tonsils (%)				< 0.001

I	43.8	33.3	54.2	
II	43.8	54.2	33.3	
III	6.3	12.5	0.0	

Classification of pharyngeal tonsil size (%)				< 0.001

Atrophic	50.8	21.0	80.5	
Normal	32.6	45.6	19.5	
Moderate hypertrophy	14.6	29.2	0.0	
Severe hypertrophy	2.1	4.2	0.0	

The data is presented as mean ± standard deviation and percentages.

According to the data collected, the present study demonstrates that, upon comparing the two groups, nasal obstruction (*p* = 0.14) had a higher trend in the group with learning difficulties (group I). The presence of apnea and nasal discharge was also higher in this group. Regarding snoring, the results were not statistically consistent to characterize the association with learning disability (*p* > 0.5).

The results pertaining to the size of the pharyngeal tonsil, based on nasopharynx radiography are shown on Table 1. They show a highly consistent relationship between learning disability and pharyngeal tonsil hypertrophy (*p* < 0.001).

The study of the percentage ratios pertaining to the values of the tonsils, using the Mann-Whitney test, showed a consistent relationship between tonsil size and learning disability (*p* < 0.001). Regarding the size of the nasal cavities, its association was not consistent (*p* = 0.75) (Table 1).

## DISCUSSION

With the intent of showing the relationship between learning disability and mouth breathing, we tried to assess, in a comparative study, children between 8 and 12 years with and without learning disabilities within 24 months.

Despite the few studies associating learning disabilities and nasal breathing, there is a consensus that apnea and nocturnal hypoxemia occur in children with hypertrophic pharyngeal and palatine tonsils, with the degree of systemic involvement directly related to the degree of tonsil hypertrophy. Complications related to blood oxygen desaturation can cause systemic repercussions, but these are not frequently reported^8^.

In 2006, in Poland, Kurnatowski et al.^9^ analyzed the influence of adenotonsillar hypertrophy in the cognitive abilities of 221 children. The authors concluded that the apnea caused by tonsil hypertrophy can lead to cognitive impairment, such as memory, concentration, attention, learning disability, low perception and sensorimotor integration. In children aged 10 to 13 years, the authors stated that memory deficits and learning disabilities are more severe in patients with adenotonsillar hypertrophy.

The relationship between obstructive sleep disorder and learning dysfunction has been well documented in studies such as Uema et al.^10^ in 2007, in which children 6-12 years of age, were divided into three groups (with sleep apnea, primary snoring and control) and evaluated by means of learning tests. Children with obstructive sleep disorder had worse results in the learning tests when compared to controls.

A study by Petry et al.^11^ in Rio Grande do Sul, in 2008, confirms the relationship between children with excessive daytime sleepiness and increased risk of habitual snoring, apnea, mouth breathing and learning problems.

In a study by Abreu et al.^12^ carried out with children from Abaeté in 2008, they assessed the main causes of mouth breathing, with allergic rhinitis coming first and adenoid hypertrophy as the second leading cause. Gozal & Kaditis^13^ in a study carried out in 2011 on possible treatments for obstructive sleep apnea, list adenotonsillar hypertrophy as a major cause of nasal obstruction, which causes obstructive sleep apnea and consequent low school performance.

In a study carried out in 2011, Bourke et al.^14^ assessed children with respiratory disorders during sleep and children with normal breathing. Intellectual skills were found to be lower in the group of children with respiratory disorders during sleep. Higher rates of difficulties in executive and school functions were also found in this group of children.

In this study, according to the interview data, 54.2% of the students with learning disabilities reported nasal obstruction. This result suggests a trend of higher prevalence of nasal obstruction in the group of children with learning disability (*p* = 0.14) - which corroborates findings in the literature. To clarify the possible causes of this obstruction, we evaluated the internal volume of the nasal cavities, the size of tonsils and adenoids. The result of this study was highly significant for the association between the degree of pharyngeal tonsil growth (*p* < 0.001) and/or palatine tonsil growth (*p* < 0.001) with learning disabilities, which also corroborates the findings of the above mentioned papers. However, in a study carried out in Iran with 320 children from the fourth grade of elementary schools, the authors used a questionnaire to assess patterns, sleep-related problems and school performance (math, science, reading, speaking and writing) and found no association between tonsil size and school performance^15^.

In our assessment of the nasal cavity volume by acoustic rhinometry, the results have not shown a consistent relationship between the two groups in the sum of the average volume of the nasal cavities (*p* = 0.75) when the two groups were compared.

Among the causes of nasal obstruction in these students, the pharyngeal tonsil hypertrophy is the primary, followed by the palatine tonsils and then by the turbinates, despite the groups having ages ranging from 8 to 12 years - age greater than expected to yield nasal obstruction or pharyngeal tonsil hypertrophy. It is likely that the pharyngeal tonsils have been hypertrophic for a long time, and it is possible that they have had it since their first childhood. The tonsils and adenoid enlargements may have caused chronic hypoxemia and consequent impairment of their cognitive and learning schemes.

Although the authors claim that pO_2_ and pCO_2_ changes exist, we know that pH does not change much in children, for they can easily compensate their acid-base balance^5^. However, other studies show that severe changes in pH, which probably occurred because of the long time and/or intensity of the mechanism that led to airway obstruction, may have been the cause of involvement of the cardiopulmonary system, and the reason for such involvement was because their compensatory systems were exhausted^16^, ^17^.

Despite the poor documentation on how nasal obstruction, in its various degrees, interfere or not with learning, it is known that relative hypoxemia (pO_2_ of approximately 72 mmHg) would not be physiologically significant^5^. The degree of involvement of this relative hypoxemia, vis-à-vis the cognitive systems of students in their learning period, besides not being detected, is not well understood, it is difficult to stipulate the critical limits for oxygen levels that can affect the central nervous system.

The set of results obtained here suggests that nasal obstruction can cause an impact on cognitive systems.

Due to the complexity of these associations, we do not intend to exhaust the subject matter in this study. It is believed that this was just another step and a warning to call the attention of the educational community concerning the need for respiratory assessment in children with learning difficulties.

## CONCLUSION

Based on this study, we concluded that children with learning disabilities have a higher prevalence of hypertrophic tonsils and adenoids when compared with children of normal school performance.
